# Machine Learning Prediction of ICU Mortality and Length of Stay in Atrial Fibrillation: A MIMIC-IV/MIMIC-III Study

**DOI:** 10.3390/healthcare14030356

**Published:** 2026-01-30

**Authors:** Victoria Nguyen, Rahul Mittal

**Affiliations:** Department of Health Informatics, Rutgers University, Piscataway, NJ 08854, USA

**Keywords:** atrial fibrillation, intensive care unit, critical care, machine learning, predictive modeling, mortality, length of stay, electronic health records, MIMIC-IV, MIMIC-III, model interpretability, SHAP

## Abstract

**Background**: Atrial fibrillation (AF) is common among intensive care unit (ICU) patients and is associated with increased mortality, prolonged length of stay (LOS), and greater resource utilization. Widely used AF risk scores were developed for stable outpatient populations and have limited applicability in critically ill patients. This study aimed to (1) characterize ICU patients with AF, (2) develop and temporally externally validate machine learning models to predict ICU mortality and ICU LOS, and (3) identify early clinical factors associated with these outcomes using interpretable methods. **Methods**: Adult ICU patients with AF from MIMIC-IV (n = 20,058) were used for model development with grouped cross-validation, and MIMIC-III (n = 11,475) served as a temporal external validation cohort. Predictors included demographics, admission characteristics, vital signs, laboratory values, vasoactive support, and AF-related medications available within the first 24 h of ICU admission. Eight classification algorithms were evaluated for ICU mortality, and six regression algorithms were evaluated for ICU LOS. Discrimination was primarily assessed using the area under the receiver operating characteristic curve (AUC) and average precision (AP), with additional threshold-dependent metrics reported to characterize operating-point behavior under low event prevalence. Probability-threshold optimization using out-of-fold predictions was applied to the primary mortality model. LOS performance was evaluated using mean absolute error (MAE), root mean squared error (RMSE), and the coefficient of determination (R^2^). Model interpretability was assessed using SHapley Additive exPlanations (SHAP). **Results**: The median age was 75 years, and ICU mortality was 8.9%. For mortality prediction, the XGBoost model demonstrated preserved discrimination on temporal external validation (MIMIC-III) (AUC = 0.743; AP = 0.226). At the default probability threshold (0.50), recall and F1 scores were low due to low event prevalence; applying a prespecified F1-optimized threshold derived from the development cohort improved sensitivity while maintaining overall discrimination. For ICU LOS, models explained little variance on temporal validation; LightGBM performed best, but the explained variance was low (MAE = 88.9 h; RMSE = 163.9 h; R^2^ = 0.038), indicating that the first 24-h structured data provide an insufficient signal to accurately predict ICU LOS, likely due to downstream clinical and operational factors. SHAP analysis identified clinically plausible predictors of mortality and prolonged ICU stay, including reduced urine output, renal dysfunction, metabolic derangement, hypoxemia, early vasopressor use, advanced age, and admission pathways.

## 1. Introduction

Atrial fibrillation (AF) is the most common sustained arrhythmia in clinical practice and is especially important in the intensive care unit (ICU) [1]. In critically ill patients, rapid and disorganized atrial activity can lead to hemodynamic instability, thromboembolism, and worsening organ dysfunction [2]. AF may be present before admission or may develop as new-onset atrial fibrillation (NOAF) during acute illness, often in the setting of sepsis, major surgery, respiratory failure, electrolyte abnormalities, or systemic inflammation [2,3,4]. Reported ICU incidence ranges from approximately 5% to more than 40%, with higher rates observed in cardiac surgical and septic cohorts [3,4,5].

ICU patients with AF consistently experience worse outcomes than those without AF. Prior studies have shown higher mortality, prolonged ICU and hospital length of stay (LOS), greater use of organ-support therapies, and increased healthcare resource utilization in this population [3,4,6]. These risks are further amplified by common comorbidities such as hypertension, diabetes, renal dysfunction, and heart failure [7,8]. Despite the clear clinical impact, early risk stratification in AF remains challenging. Widely used outpatient-based scoring tools, such as CHA_2_DS_2_-VASc, were not designed for critically ill patients and have limited applicability in the ICU [9].

With the growth of electronic health record (EHR) systems, large volumes of ICU data are now routinely available, including demographics, vital signs, laboratory results, comorbidities, and details of organ-support therapies. This has enabled the use of machine learning (ML) for outcome prediction in AF. Recent studies have applied ML to predict NOAF in critically ill patients [5], to estimate short-term mortality among ICU patients with AF and comorbid conditions such as hypertension or heart failure [10,11,12], and in some cases to outperform traditional clinical scores [9]. Algorithms including random forests, gradient boosting methods, XGBoost, and LightGBM have been used to capture complex, nonlinear relationships between physiology, treatments, and outcomes [5,11,12]. In parallel, interpretability techniques such as SHapley Additive exPlanations (SHAP) have made it possible to identify which clinical features drive model predictions, supporting potential bedside adoption of ML tools [13,14].

Recent advances in deep learning have shown promise for modeling complex clinical data, including applications that leverage longitudinal and high-dimensional electronic health record information for outcome prediction in critical care and atrial fibrillation populations [13,14]. At the same time, explainability has emerged as a central challenge for clinical deployment of deep learning models, particularly when applied to complex, temporally evolving data [15]. Many such approaches require dense data streams, substantial computational resources, and tightly integrated electronic health record pipelines, and their outputs may be less transparent to clinicians than tree-based models paired with established interpretability frameworks. Because the present study focuses on early risk stratification using routinely available structured variables from the first 24 h of ICU admission, we prioritized gradient-boosting models that perform well on tabular data and can be interpreted using SHAP to support clinical face validity and reproducibility [13,14,15].

Accordingly, tree-based ensemble methods such as gradient boosting and XGBoost remain widely used in clinical prediction tasks because they perform well on structured tabular data, are robust to missingness and nonlinear interactions, and can be paired with interpretable frameworks such as SHAP to identify clinically meaningful predictors. For early ICU risk stratification based on routinely available data from the first 24 h of admission, these models offer a practical balance between predictive performance, transparency, and feasibility for clinical decision support.

However, important gaps remain in this literature. First, many existing ML models focus on specific subgroups (for example AF with heart failure or AF in selected high-risk cohorts), rather than on a broad AF ICU population. Second, most work has emphasized in-hospital or 30-day mortality, while LOS has received far less attention, even though AF is clearly associated with prolonged ICU stays and higher resource use [4,6,16]. Third, external validation is rare, and few models provide both strong performance and interpretable explanations that clinicians can use to understand risk drivers in AF patients.

The present study aims to address these gaps by leveraging the Medical Information Mart for Intensive Care databases (MIMIC-IV for model development and MIMIC-III for temporal external validation). Specifically, we seek to:

1. Characterize the demographics, comorbidities, and early clinical features of ICU patients with AF;
2. Develop and validate machine learning models using a temporal external validation cohort (MIMIC-III) to predict ICU mortality and LOS using data from the first 24 h of ICU admission;
3. Identify key clinical predictors of adverse outcomes using SHAP-based interpretability.

By combining large-scale ICU data with interpretable machine learning methods, this study aims to improve early prognostication, highlight clinically important risk factors, and support more informed decision-making for the management of atrial fibrillation in critical care settings.

## 2. Materials and Methods

### 2.1. Study Design and Data Sources

This was a retrospective cohort study using data from the Medical Information Mart for Intensive Care IV (MIMIC-IV, version 3.1) and MIMIC-III (version 1.4), two large, publicly available, de-identified ICU databases developed by the Massachusetts Institute of Technology in collaboration with Beth Israel Deaconess Medical Center. MIMIC-IV contains ICU admissions from 2008 to 2019, and MIMIC-III covers 2001 to 2012. Both databases include detailed information on demographics, vital signs, laboratory results, medications, procedures, and outcomes for critically ill patients.

All required human subjects research training was completed (CITI Certification ID: 68788415), and access was obtained through the PhysioNet credentialed data use process. Because all data are de-identified, additional institutional review board approval was not required.

MIMIC-IV was used for model development and internal validation, and MIMIC-III served as a temporal external validation cohort. Although MIMIC-III represents an independent dataset with a distinct time period and database structure, both MIMIC-IV and MIMIC-III originate from the same academic medical center; therefore, this validation should be interpreted as temporal external validation rather than full multicenter generalizability.

### 2.2. Study Population and Outcomes

The study cohort included adult patients (≥18 years) who were admitted to the ICU with a diagnosis of atrial fibrillation identified from hospital discharge codes at the admission level. In MIMIC-IV, atrial fibrillation was defined using ICD-9 code 42,731 and ICD-10 codes I480, I481, I482, and I4891, corresponding to paroxysmal, persistent, long-standing persistent, chronic or permanent, and unspecified atrial fibrillation. In MIMIC-III, atrial fibrillation was identified using ICD-9 code 42,731 only. Atrial flutter (ICD-9 42,732) was excluded in both databases.

For each hospital admission with atrial fibrillation, only the first ICU stay was retained as the index episode. ICU stays shorter than 24 h were excluded to ensure complete 0–24 h feature availability anchored at ICU admission (T0). A single analysis row was created for each ICU stay, identified by stay_id (MIMIC-IV) or icustay_id (MIMIC-III). The variables subject_id and hadm_id were retained only for grouping and to prevent information leakage during cross-validation, not as model features.

Age in MIMIC-IV was calculated using the anchor-based de-identification schema. In MIMIC-III, ages above 89 years were handled according to the public de-identification rules. Patients with calculated age <18 years, implausible or inconsistent timestamps (for example, ICU discharge time less than or equal to ICU admission time), or missing outcome labels were removed.

Two outcomes were defined at the level of each ICU episode. For the classification task, the outcome was ICU mortality, defined as death occurring during the index ICU stay. For the regression task, the outcome was ICU LOS, calculated in hours as the time between ICU admission and ICU discharge for the same episode. LOS was measured as continuous hours (not rounded), consistent with the ICU timestamp resolution in MIMIC. All cross-validation procedures and data splits were grouped by subject_id so that all episodes from a given patient were kept in the same fold, ensuring that no individual patient contributed data to both the training and validation sets and preventing information leakage.

The final cohorts included 20,058 ICU patients in MIMIC-IV and 11,475 ICU patients in MIMIC-III.

### 2.3. Predictors and Feature Extraction

Predictor variables were derived to reflect information available within the first 24 h after ICU admission. Demographic and admission variables were extracted from the patients and admissions tables and included age at admission, sex, race or ethnicity, primary language, marital status, insurance type, admission type (emergency, elective, urgent), and admission location (for example, emergency department, ward, and operating room).

Physiologic and laboratory features were restricted to clinically interpretable extremes within the 0-to-24-h window. Vital signs included minimum mean arterial pressure, minimum oxygen saturation, maximum heart rate, maximum respiratory rate, and minimum and maximum temperature. Laboratory features included minimum hemoglobin, minimum platelet count, maximum white blood cell count, minimum albumin, maximum anion gap, minimum bicarbonate, maximum blood urea nitrogen, maximum creatinine, minimum and maximum serum glucose, minimum and maximum sodium, minimum and maximum potassium, minimum chloride, minimum calcium, and total urine output over the first 24 h.

Organ support and atrial fibrillation-related medications were represented as binary indicators capturing any exposure during the 0-to-24-h window. These included renal replacement therapy and vasoactive or inotropic agents (norepinephrine, epinephrine, phenylephrine, vasopressin, dopamine, dobutamine, angiotensin II, and milrinone). Commonly used antithrombotic and antiplatelet agents and rate- or rhythm-control drugs were also flagged, including heparin, aspirin, warfarin, metoprolol, amiodarone, diltiazem, digoxin, esmolol, labetalol, and propranolol. Medication flags were derived from prescription start and stop times that overlapped the interval from T0 to T0 plus 24 h.

The same 0-to-24-h predictors were used for both the ICU mortality classification and ICU LOS regression tasks in MIMIC-IV and MIMIC-III.

### 2.4. Data Preprocessing and Handling of Missing Values

All preprocessing was implemented using reproducible scikit-learn pipelines to avoid information leakage and to ensure consistent transformations across internal and external datasets. Identifier columns (subject_id, hadm_id, stay_id, icustay_id, and timestamp fields) and outcome labels were excluded from the feature matrix but retained as grouping keys.

Before modeling, all numeric fields that were stored as text were converted to floating-point numbers. For continuous (numeric) predictors, missing values were imputed using the median of each variable calculated from the training data only, and then each numeric feature was standardized to have mean 0 and standard deviation 1 using StandardScaler. For categorical predictors, missing values were imputed with the most frequent category (mode) observed in the training data, and categories were then transformed using one-hot encoding with handle_unknown = “ignore” to allow for unseen levels in the validation and external datasets.

All of these preprocessing steps were implemented within a single scikit-learn ColumnTransformer and pipeline. The transformer was fitted only on the training portion of each cross-validation fold, and the learned imputation, scaling, and encoding parameters were then applied unchanged to the corresponding validation folds and to the external MIMIC-III cohort, preventing information leakage and ensuring consistent feature representation across datasets.

To ensure feature alignment between MIMIC-IV and MIMIC-III, any predictors present in MIMIC-IV but absent in MIMIC-III were added to the external dataset and filled with missing values before applying the same preprocessing pipeline. This step guaranteed identical column order and prevented errors due to column mismatches.

### 2.5. Model Development

Two supervised learning problems were addressed: ICU mortality as a binary classification task and ICU LOS as a continuous regression task. All models were implemented in Python 3.12.12 using scikit-learn 1.6.1, XGBoost 3.1.3, and LightGBM 4.6.0 libraries.

For mortality prediction, multiple classifiers were trained on the MIMIC-IV cohort, including logistic regression, decision trees, random forests, gradient boosting (scikit-learn implementation), AdaBoost, k-nearest neighbors, multilayer perceptron, and an XGBoost classifier. Five-fold StratifiedGroupKFold cross-validation was used, stratified by the mortality outcome and grouped by subject_id to prevent patient-level leakage. Within each fold, the preprocessing pipeline was fitted on the training subset only, and the transformed features were passed to the classifier and evaluated on the held-out fold. Out-of-fold predicted probabilities from all folds were concatenated to summarize internal performance and to support probability-threshold optimization.

For ICU LOS prediction, analogous preprocessing and grouped cross-validation were applied using several regression algorithms: linear regression, random forest, gradient boosting (scikit-learn), k-nearest neighbors, multilayer perceptron, and a LightGBM regressor. GroupKFold with grouping by subject_id ensured that all ICU episodes from a given patient appeared in only one fold.

Because ICU LOS was right-skewed, we evaluated both untransformed LOS (hours) and a log1p-transformed LOS as a sensitivity analysis. Models trained on log1p were back-transformed to hours for evaluation. As log1p models did not improve transportability on temporal external validation (MIMIC-III), primary LOS performance results are reported for the untransformed (hour-scale) models.

For interpretability, SHAP analyses for ICU LOS were computed using the hour-scale LightGBM model to align feature-attribution results with the primary LOS models and reported performance metrics.

**Figure 1:**
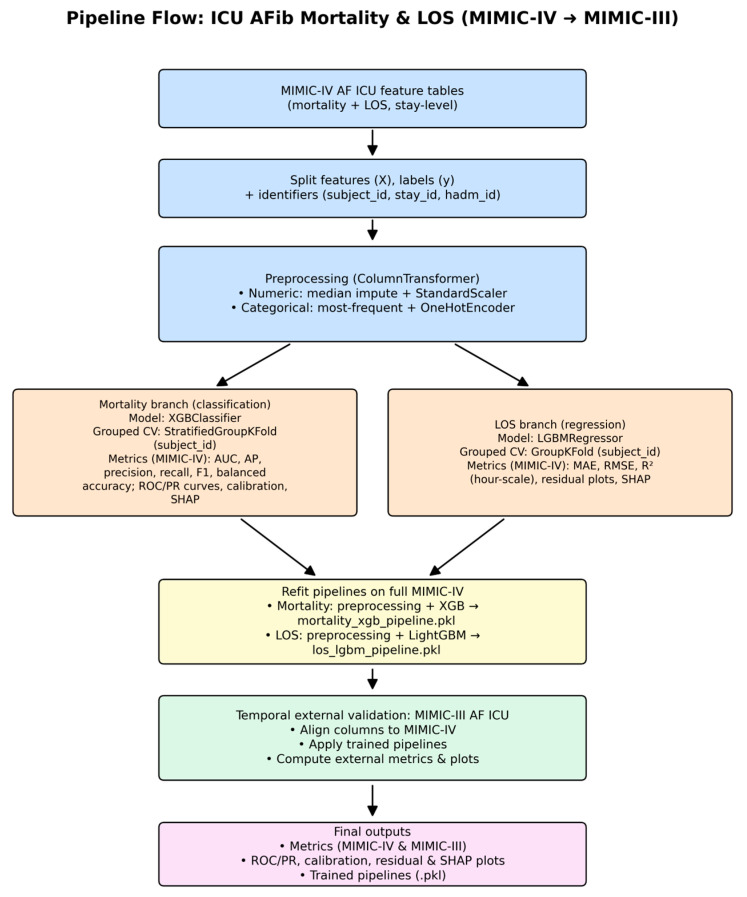
Pipeline flow for ICU atrial fibrillation mortality and LOS models (MIMIC-IV to MIMIC-III). Note: AF: atrial fibrillation, ICU: intensive care unit, LOS: length of stay, MIMIC: Medical Information Mart for Intensive Care, XGB: Extreme Gradient Boosting, LGBM: Light Gradient Boosting Machine, CV: cross-validation, AUC: area under the receiver operating characteristic curve, AP: average precision, MAE: mean absolute error, RMSE: root mean squared error, R^2^: coefficient of determination, ROC: receiver operating characteristic, PR: precision–recall, SHAP: SHapley Additive exPlanations, PKL: pickle (Python serialized model file).

Based on internal cross-validation and temporal external validation, the primary mortality model was an XGBoost pipeline, and the primary LOS model was a LightGBM regressor. These models were then refitted on the full MIMIC-IV development dataset before being evaluated on the corresponding MIMIC-III external cohorts. Figure 1 summarizes the end-to-end modeling pipeline from cohort construction through internal and temporal external validation for both ICU mortality and LOS.

Hyperparameter selection followed a pragmatic strategy aligned with clinical deployment considerations. For most scikit-learn baseline models, default or minimally adjusted parameters were used. For the primary XGBoost and LightGBM models, prespecified parameter values (including number of estimators, learning rate, tree depth or number of leaves, and subsampling rates) were chosen as reasonable settings and assessed through grouped cross-validation performance in the development cohort. Extensive grid search or Bayesian hyperparameter optimization was intentionally not performed to avoid optimistic bias and to reflect realistic clinical model development settings.

#### Handling of Class Imbalance

ICU mortality was a relatively rare outcome in the development cohort (approximately 9% prevalence in MIMIC-IV). To account for class imbalance during model training and evaluation, several complementary strategies were employed.

First, performance metrics that are robust to class imbalance, including the area under the receiver operating characteristic curve and the area under the precision–recall curve, were prioritized over raw accuracy. Balanced accuracy, sensitivity, specificity, precision, and F1 score were also reported to provide complementary perspectives on classification performance.

Second, algorithm-specific weighting strategies were applied during model training where supported. For example, class-weighted loss functions were used for logistic regression, random forest, and decision tree models, and the scale_pos_weight parameter was used for XGBoost to upweight the minority (mortality) class.

Third, for the primary mortality model (XGBoost), additional sensitivity analyses were conducted to assess the robustness of model performance under alternative imbalance-handling strategies. These included (1) negative-class downsampling to achieve a balanced training set and (2) combined synthetic oversampling and Tomek link removal (SMOTETomek). These approaches were applied only to the primary model to evaluate the impact of resampling on discrimination and calibration, rather than to optimize performance through aggressive oversampling.

Model discrimination and calibration were compared across these strategies using out-of-fold predictions and temporal external validation on MIMIC-III. The results demonstrated stable discrimination across imbalance-handling approaches, supporting the robustness of the modeling framework.

### 2.6. Model Evaluation and Statistical Analysis

Because the project included both classification (mortality) and regression (LOS) outcomes, separate metric sets were applied.

For the classification task, ICU mortality was a relatively rare outcome, and class imbalance rendered raw accuracy difficult to interpret. Accordingly, performance was summarized primarily using threshold-independent metrics, including the area under the receiver operating characteristic curve (AUC) and the area under the precision–recall curve (AP). Threshold-dependent metrics, sensitivity (recall), specificity, precision, F1 score, accuracy, and balanced accuracy, were also reported based on confusion matrices computed on held-out data.

Class imbalance was addressed during model development through class-weighting strategies (Section Handling of Class Imbalance). In addition, to support clinically meaningful decision-making at deployment, an explicit probability-threshold analysis was performed for the primary mortality model. Using out-of-fold predictions from grouped cross-validation in the development cohort (MIMIC-IV), probability thresholds ranging from 0.01 to 0.99 were evaluated to characterize precision–recall trade-offs under low event prevalence. A single prespecified threshold optimized for F1 score was selected a priori as a pragmatic operating point balancing sensitivity and precision.

This F1-optimized threshold (approximately 0.17–0.18 across analyses) was applied unchanged to the temporal external validation cohort (MIMIC-III). For transparency, performance of the primary XGBoost model is reported at both the default probability cutoff (0.50) and the prespecified F1-optimized cutoff to illustrate threshold-dependent trade-offs under low event prevalence. All other classification models are reported at the default threshold (0.50) to allow consistent algorithmic comparison. Threshold-dependent results for all candidate models are provided in the Supplementary Materials (Table S1). Balanced accuracy was included as a prevalence-robust summary metric, complementing discrimination measures and facilitating interpretation under imbalanced outcome distributions.

For the regression task, predictive accuracy for ICU LOS was summarized using mean absolute error, root mean squared error, and the coefficient of determination. Lower error values and higher R-squared values indicated better fit. As a sensitivity analysis, LOS models were also trained on log-transformed outcomes (log1p), with predictions back-transformed to the original scale for evaluation.

Descriptive analyses were conducted to compare baseline characteristics between survivors and non-survivors of the index ICU stay (y_icu_mortality: 0 = survived, 1 = died). All analyses were performed in Python using pandas, NumPy, SciPy, and scikit-learn in Jupyter Notebook 6.5.7. Categorical variables were summarized as counts and percentages and compared using Pearson chi-squared tests, with Fisher exact tests used when expected cell counts were less than 5. Continuous variables were summarized as medians with interquartile ranges and compared using the Mann–Whitney U test. Two-sided *p*–values less than 0.05 were considered statistically significant.

Before analysis, the development and validation datasets were cleaned to convert string-encoded numerics, align columns between MIMIC-IV and MIMIC-III, and apply the imputation strategies described above for missing values. Identifier variables (subject_id, hadm_id, stay_id, and icustay_id) were excluded from statistical summaries and hypothesis tests.

### 2.7. Software Environment and Reproducibility

All data extraction, preprocessing, modeling, and validation were conducted in Python version 3.12.12. Data wrangling used pandas and NumPy, and all machine learning models were implemented with scikit-learn 1.6.1, XGBoost 3.1.3, and LightGBM 4.6.0. SHAP was used for model explainability, and figures were generated with Matplotlib 3.10.0. Cohort construction and feature assembly for MIMIC-IV and MIMIC-III were performed using Google BigQuery (Standard SQL), and query outputs were exported as comma-separated-value (CSV) files for downstream analysis.

## 3. Results

### 3.1. Patient Characteristics

**Figure 2:**
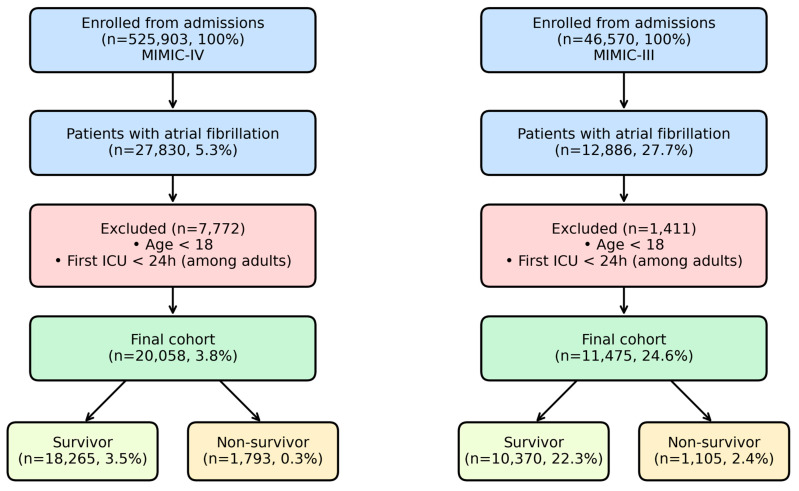
Flow diagram illustrating patient inclusion in the MIMIC-IV development cohort and MIMIC-III external validation cohort. Note: ICU: intensive care unit, MIMIC: Medical Information Mart for Intensive Care.

The final MIMIC-IV development cohort included 20,058 ICU patients with atrial fibrillation, of whom 18,265 (91.1%) survived the index ICU stay and 1793 (8.9%) died. Figure 2 illustrates the flow of patient selection for the MIMIC-IV development cohort and the MIMIC-III external validation cohort. Baseline characteristics grouped by ICU survival status are presented in Table 1.

Non-survivors were older than survivors (median 77 vs. 75 years, *p* < 0.001) and had a slightly higher proportion of female patients (43.6% vs. 40.1%, *p* = 0.004). Racial distributions differed significantly between the groups: non-survivors had a larger proportion of “Unknown” race (18.6% vs. 10.6%) and a smaller proportion of White patients (65.8% vs. 73.7%). English remained the primary language for most patients, and although non-English speakers were slightly more common among non-survivors (11.0% vs. 9.7%), this difference was not statistically significant. Medicare coverage predominated in both groups but was more frequent among non-survivors (78.0% vs. 74.2%), whereas private insurance was less common (12.7% vs. 16.6%) (*p* < 0.001).

Patterns of hospital admission also differed. Non-survivors were more likely to be admitted through the emergency department (57.4% vs. 48.1%) or transferred from another hospital (37.0% vs. 28.4%) or skilled nursing facility (2.7% vs. 2.2%), while elective and surgical same-day admissions were more common among survivors (both *p* < 0.001). In addition, survivors had a higher proportion of physician referral or clinic admissions (30.6% vs. 16.0%).

Vital signs and laboratory values from the first 24 h of ICU stay demonstrated greater physiological instability among non-survivors. These patients had lower minimum MAP (54 vs. 58 mmHg), lower minimum SpO_2_ (91% vs. 92%), and higher maximum heart rate (114 vs. 101 beats/min). Laboratory abnormalities included higher WBC, BUN, and creatinine values, lower bicarbonate levels, and markedly lower urine output (median 880 vs. 1460 mL), all *p* < 0.001. These differences reflect more severe hemodynamic compromise, metabolic derangements, and renal dysfunction among non-survivors.

Differences in early medication and vasoactive therapy were also pronounced. Norepinephrine use was more than twice as common in non-survivors (42.8% vs. 16.2%), as was vasopressin use (18.5% vs. 3.9%), and over half of non-survivors required at least one vasoactive or inotropic agent during the first 24 h compared with 38.8% of survivors (*p* < 0.001). Non-survivors were also more likely to receive heparin, amiodarone, and digoxin, while survivors more commonly received aspirin and metoprolol formulations.

Overall, the cohort reflects an older ICU population with a high burden of acute illness, where non-survivors exhibited more urgent admission routes, significantly worse early physiologic and laboratory profiles, greater use of vasoactive support, and distinct medication patterns compared with survivors.

### 3.2. Performance of Classification Models for ICU Mortality

Table 2 summarizes the temporal external validation performance of all candidate ICU mortality classifiers on the MIMIC-III cohort. Discrimination was assessed using the area under the receiver operating characteristic curve (AUC) and average precision (AP), which are independent of probability thresholds. Threshold-dependent metrics were computed using prespecified probability thresholds. For the primary XGBoost model, an F1-optimized threshold was derived from out-of-fold predictions in the MIMIC-IV development cohort and applied unchanged to MIMIC-III. All other models were evaluated at the default threshold (0.50).

At the default probability threshold of 0.50, all classifiers, including XGBoost, exhibited very low recall and F1 scores despite moderate AUC values. This behavior reflects the low mortality prevalence in the external cohort (<10%), under which a high threshold leads models to predict very few deaths. Consequently, accuracy was dominated by correct classification of survivors and was not considered clinically informative under this setting.

To address this limitation, an explicit probability threshold optimization was conducted for the primary XGBoost model using out-of-fold predictions from the MIMIC-IV development cohort. The F1-optimized threshold (approximately 0.17–0.18 across analyses) was applied unchanged to the temporal external MIMIC-III cohort. At this threshold, XGBoost achieved improved sensitivity and F1 score while preserving overall discrimination (AUC = 0.743, AP = 0.226), demonstrating a more clinically meaningful trade-off between false positives and false negatives under low event prevalence.

Across all candidate models evaluated at the default threshold (Supplementary Table S1), XGBoost demonstrated the strongest overall discrimination on temporal external validation (AUC = 0.743, AP = 0.226). Random forest and gradient boosting achieved similar AUC values but produced very few positive predictions at the default cutoff, resulting in near-zero F1 scores. Logistic regression identified a greater number of deaths at the default threshold but with lower overall discrimination. The multilayer perceptron generated substantially more positive predictions, yielding higher recall and F1 at the default threshold, but at the expense of reduced accuracy and discrimination.

**Figure 3:**
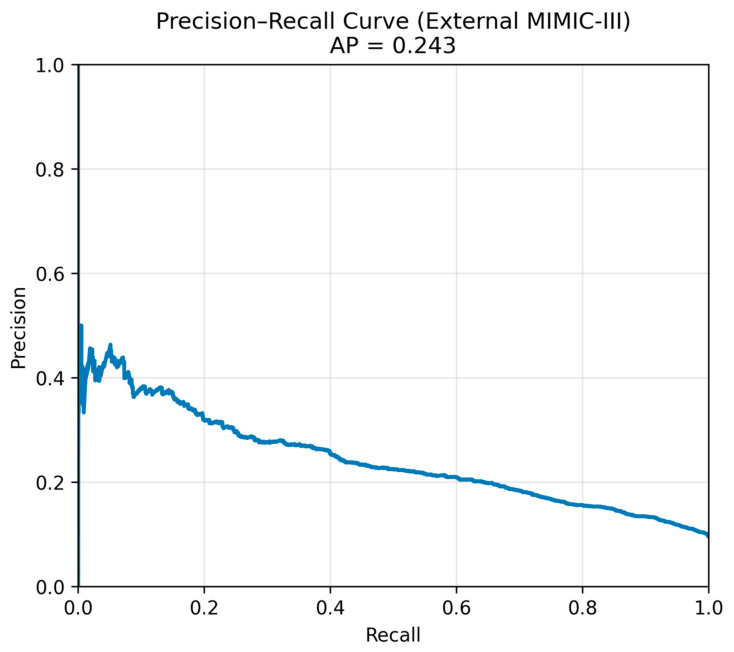
Precision–recall curve for the XGBoost model on the temporal external MIMIC-III cohort. Note: AP: average precision, MIMIC-III: Medical Information Mart for Intensive Care III.

**Figure 4:**
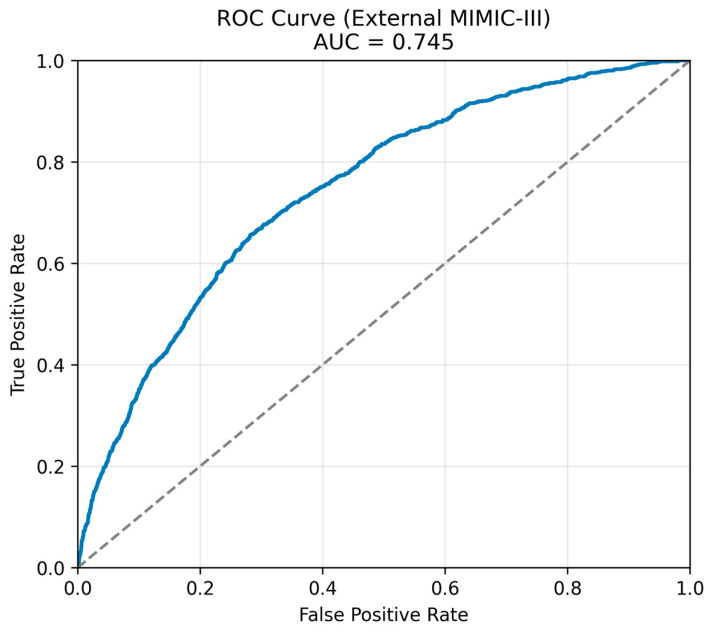
Receiver operating characteristic curve for the XGBoost model on the temporal external MIMIC-III cohort. The dashed diagonal line represents the line of no discrimination (AUC = 0.5). Note: AUC: area under the receiver operating characteristic curve, ROC: receiver operating characteristic, MIMIC-III: Medical Information Mart for Intensive Care III.

Figure 3 presents the precision–recall curve for the XGBoost model on the temporal external MIMIC-III cohort, demonstrating an average precision of approximately 0.23, consistent with the values reported in Table 2. Precision remained consistently above the baseline mortality prevalence across a wide range of recall values, indicating effective risk ranking despite substantial class imbalance. Figure 4 shows the corresponding receiver operating characteristic curve, with an external AUC of 0.743, demonstrating preserved discrimination when evaluated on an independent cohort.

**Figure 5:**
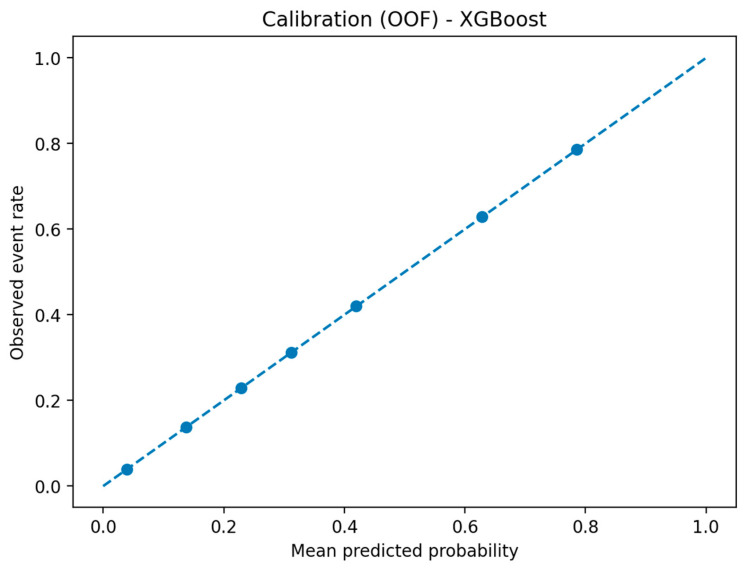
Calibration curve for the XGBoost mortality model on the temporal external MIMIC-III cohort. OOF indicates out-of-fold predicted probabilities derived from cross-validation.

Figure 5 displays the calibration curve for the XGBoost model on the temporal external MIMIC-III cohort. The model tended to underestimate absolute mortality risk at lower predicted probabilities, although relative risk ordering remained reasonable. This pattern suggests that additional recalibration would be required before deployment for absolute risk estimation in clinical settings.

### 3.3. Performance of Regression Models for ICU Length of Stay

Table 3 summarizes the performance of regression models for predicting ICU LOS on the MIMIC-III temporal external cohort. Across models, discrimination of ICU LOS was modest, with small but positive improvements over a baseline mean predictor for several algorithms, highlighting both the presence of early clinical signal and the substantial influence of downstream operational factors on LOS.

Although log1p transformation improved apparent fit during internal cross-validation, it did not improve and often worsened performance on temporal validation in MIMIC-III (negative R^2^ in several models after back-transformation). Therefore, we retained hour-scale LOS models for primary reporting.

SHAP interpretability results for LOS (Section 3.5.2) are presented for the hour-scale LightGBM model to align feature attributions with the primary LOS models and reported performance metrics.

Among the candidate models, LightGBM showed the most favorable trade-off between prediction error and explained variance, although overall performance remained modest, with MAE of 88.9 h, RMSE of 163.9 h, and R^2^ of 0.038. Gradient Boosting showed similar error metrics but a slightly lower R^2^. Linear regression produced comparable RMSE with a higher MAE and similar R^2^. Random Forest and KNN had negative R^2^ values and higher RMSE, indicating poorer generalization than simply predicting the mean ICU LOS. The MLP model performed worst, with substantially higher error and a strongly negative R^2^, consistent with poor generalization to the external cohort.

The increasing dispersion of residuals at longer LOS values likely reflects variability driven by non-physiologic factors such as bed availability, discharge planning, and post-ICU care coordination. External diagnostic plots of predicted versus observed ICU LOS and residual distributions (Supplementary Materials, Figures S1–S3) showed that the LightGBM model tended to underestimate very prolonged stays. Residuals were right-skewed and became more dispersed as true ICU LOS increased, highlighting the difficulty of accurately modeling extreme ICU stays using first-day structured data alone.

### 3.4. Deployment of the Prediction Models as a Web Application

The final XGBoost mortality model and LightGBM length-of-stay model were deployed in an interactive web application (available at: https://huggingface.co/spaces/ICU-Afib-Lab/icu-afib-prediction (accessed on 1 January 2026)). The application serves as a proof-of-concept demonstration of how first-day ICU data may be used for early risk stratification in patients with atrial fibrillation. The interface is intended for illustrative purposes and allows users to explore model behavior under varying input values and decision thresholds. By publicly deploying models that underwent temporal external validation with identical preprocessing and inference pipelines, the application enhances transparency, reproducibility, and accessibility of the proposed approach for future methodological and clinical research.

The interface accepts entry of the 54 first-day ICU predictors used during model development, including demographics, admission characteristics, vital signs, laboratory measurements, vasoactive support, and atrial fibrillation–related medications. Based on these inputs, the application returns an estimated probability of ICU mortality and a predicted ICU length of stay. Predictions are generated using the same preprocessing pipelines and trained models applied in the internal cross-validation and temporal external validation analyses, ensuring consistency between reported performance metrics and deployed outputs.

Because the models rely on structured variables available within the first 24 h of ICU admission, the application is designed to support post-diagnosis risk stratification rather than primary diagnosis of atrial fibrillation. The current implementation requires manual data entry and is therefore not intended for direct clinical deployment. However, it illustrates the potential for integration into electronic health record systems, where automated extraction of routinely collected ICU data could enable real-time or near–real-time decision support without increasing clinician workload.

Future extensions of this platform include automated EHR data ingestion, dynamic risk updating beyond the first 24 h, and incorporation of time-series or waveform-based features with interpretable attribution methods, which may further improve predictive performance and clinical utility [17].

### 3.5. Key Clinical Factors Associated with ICU Outcomes

To characterize how first-day ICU variables relate to predicted outcomes in patients with atrial fibrillation, SHapley Additive exPlanations (SHAP) were used to interpret the fitted mortality and length-of-stay (LOS) models. SHAP values quantify the contribution of each predictor to the model output for individual patients while accounting for interactions with other variables. This approach allows identification of clinical factors most strongly associated with higher predicted ICU mortality and longer ICU stays.

#### 3.5.1. Predictors of ICU Mortality

**Figure 6:**
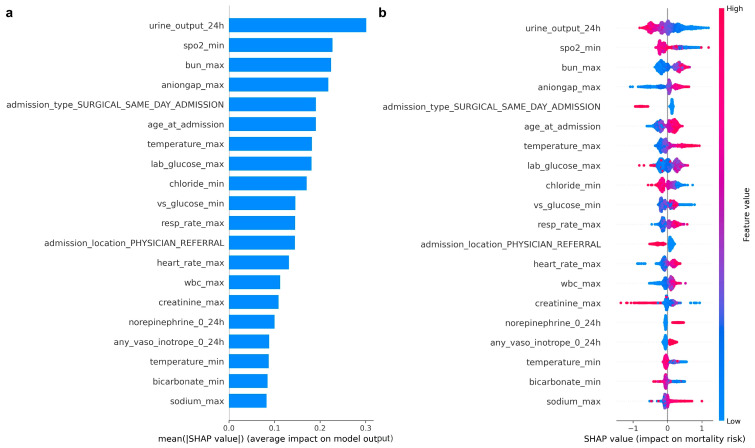
SHAP summary plots for the XGBoost ICU mortality model in the MIMIC-IV development cohort. Panel (**a**) shows mean absolute SHAP values ranking the 20 most influential predictors. Panel (**b**) shows a SHAP beeswarm plot illustrating the magnitude and direction of each feature’s effect on predicted mortality; points to the right indicate higher predicted risk and points to the left indicate lower predicted risk, with color representing higher versus lower feature values. Note: SHAP: SHapley Additive exPlanations.

After comparison of classifier performance across internal and temporal external datasets, the XGBoost pipeline was selected as the primary mortality model for feature importance analysis. Figure 6 presents the SHAP summary plots for this model using the MIMIC-IV development cohort. Panel (a) ranks predictors by their mean absolute SHAP values, while panel (b) illustrates the direction and magnitude of each feature’s association with predicted ICU mortality.

Lower 24-h urine output emerged as the most influential predictor, with reduced urine output consistently associated with increased mortality risk. Markers of respiratory and metabolic derangement, including lower minimum oxygen saturation, higher maximum blood urea nitrogen, and higher anion gap, also ranked prominently, reflecting impaired oxygenation, renal dysfunction, and metabolic stress early in the ICU course. Older age at admission and higher maximum temperature were additional strong contributors to increased predicted risk.

Several treatment- and admission-related variables further influenced mortality predictions. Exposure to norepinephrine and other vasoactive or inotropic agents within the first 24 h was associated with higher predicted mortality, consistent with early hemodynamic instability. Admission pathways, including surgical same-day admission status and physician referral, also contributed meaningfully, likely capturing differences in baseline severity, acuity, and pre-ICU care.

Overall, the SHAP patterns indicate that early physiologic instability, impaired perfusion, metabolic abnormalities, and specific admission characteristics are strongly associated with ICU mortality risk among patients with atrial fibrillation. These findings are consistent with the observed differences in baseline characteristics between survivors and non-survivors in the cohort.

#### 3.5.2. Predictors of ICU Length of Stay

**Figure 7:**
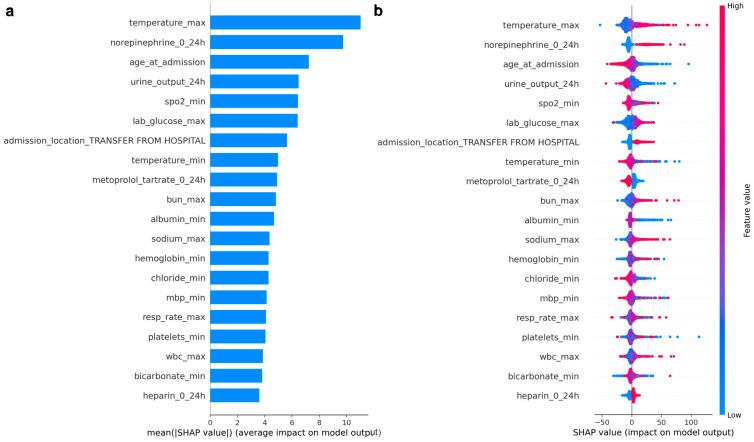
SHAP summary plots for the LightGBM ICU length of stay model in the MIMIC-IV development cohort. Panel (**a**) shows mean absolute SHAP values ranking the 20 most influential predictors. Panel (**b**) shows a SHAP beeswarm plot illustrating the magnitude and direction of each feature’s effect on predicted ICU LOS; points to the right indicate longer predicted stays and points to the left indicate shorter predicted stays, with color representing higher versus lower feature values. Note: SHAP: SHapley Additive exPlanations.

Figure 7 presents the SHAP summary plots for the LightGBM model predicting ICU length of stay, derived from the MIMIC-IV development cohort. SHAP values quantify the contribution of each first-day ICU variable to the model’s prediction of longer or shorter ICU stays measured in hours. Panel (a) ranks predictors by their mean absolute SHAP values, reflecting overall importance, while panel (b) illustrates the direction and magnitude of each feature’s association with predicted ICU LOS.

Across the cohort, maximum temperature emerged as the most influential predictor of ICU length of stay, with higher temperatures associated with longer predicted stays. Early norepinephrine exposure within the first 24 h was also strongly associated with prolonged ICU LOS, highlighting the role of early hemodynamic instability and need for vasopressor support. Older age at admission, lower 24-h urine output, and lower minimum oxygen saturation further contributed to longer predicted stays, reflecting physiologic vulnerability, impaired perfusion, and respiratory compromise early in the ICU course.

Laboratory measures of metabolic and renal dysfunction were also prominent contributors to ICU LOS predictions. Higher blood urea nitrogen, lower serum albumin, higher serum sodium, lower hemoglobin, and lower bicarbonate were associated with longer ICU stays, suggesting that systemic illness severity, renal impairment, and metabolic imbalance play important roles in extending ICU duration. Lower mean blood pressure, higher respiratory rate, and abnormalities in white blood cell count, platelet count, and chloride further reflected the contribution of inflammatory stress and physiologic instability.

Admission and treatment-related factors also influenced LOS predictions. Admission from another hospital was associated with longer predicted ICU stays, likely reflecting greater baseline complexity or severity of illness at presentation. Early exposure to selected medications, including metoprolol and heparin, contributed modestly to LOS predictions and may reflect differences in underlying comorbidity burden, treatment strategies, or care trajectories rather than direct causal effects.

Overall, the SHAP patterns indicate that prolonged ICU length of stay among patients with atrial fibrillation is primarily associated with early physiologic derangement, need for organ support, and markers of systemic illness severity observed within the first 24 h of ICU admission. While several predictors overlap with those influencing ICU mortality, their relative importance differs, underscoring that prolonged ICU utilization reflects sustained illness burden and recovery dynamics rather than acute risk of death alone.

## 4. Discussion

In this large, retrospective cohort of ICU patients with atrial fibrillation, we developed machine learning models to predict ICU mortality and LOS using routinely available data from the first 24 h of ICU admission and validated them using a temporal external validation cohort (MIMIC-III). The main findings are threefold. First, an XGBoost classifier trained on 54 clinical predictors achieved moderate discrimination for ICU mortality in an older, high-risk AF population, with preserved performance on the temporal external MIMIC-III cohort. Although MIMIC-III represents a temporally distinct cohort, both datasets derive from a single academic center; therefore, broader multicenter validation is still required. Second, a LightGBM regressor for ICU length of stay provided only modest explanatory power, underscoring the multifactorial and non-clinical determinants of ICU duration. Third, SHAP-based interpretability identified clinically plausible risk factors, particularly early hemodynamic instability, metabolic derangement, renal dysfunction and admission pathways, as key drivers of both mortality and prolonged ICU stay.

### 4.1. Comparison with Prior Work

The ICU mortality rate in this AF cohort (8.9%) is consistent with previous studies that describe AF in the critically ill as a marker of substantial risk and resource use [1,2,3,4]. Similar to reports from large multicenter cohorts and scoping reviews, patients with AF in our study were typically older, predominantly Medicare insured and frequently admitted via emergency or urgent routes, with higher illness severity among non-survivors [3,4,5,6,16]. These baseline patterns reinforce the notion that AF in the ICU is not an isolated rhythm disorder but part of a broader syndrome of multi-organ dysfunction in vulnerable patients.

Our XGBoost mortality model, which achieved an AUC of approximately 0.75 on temporal external validation, aligns with recent work demonstrating that ensemble methods and gradient boosting algorithms can provide clinically useful discrimination for AF-related outcomes in critically ill populations [5,7,8,9,10,11,12]. Guan et al. and Alomari et al. reported comparable performance for predicting new-onset AF or AF-related complications using multicenter ICU data [5,12], while Ledziński et al. showed that machine learning models trained on MIMIC-IV can outperform CHA_2_DS_2_-VASc for mortality prediction in AF patients treated with direct oral anticoagulants [9]. Similarly, Verhaeghe et al. developed calibrated machine learning models for AF risk prediction in ICU patients and emphasized the importance of robust validation and calibration before clinical deployment [11]. Our study adds to this literature by focusing specifically on ICU mortality and LOS among AF patients, using both internal grouped cross-validation and external validation across two versions of MIMIC.

Importantly, our findings support the growing consensus that outpatient-derived risk scores, such as CHA_2_DS_2_-VASc, are not well suited for critically ill AF populations, where acute physiology and organ support dominate prognosis [2,4,9,14]. By leveraging high-resolution first-day ICU data, the present models better reflect the dynamic pathophysiology of critical illness, complementing prior work that has applied machine learning to predict ICU outcomes in AF patients with comorbid hypertension, diabetes or heart failure [7,8,10].

### 4.2. Model Interpretability and Clinical Face Validity

A key strength of this study is the emphasis on model interpretability using SHAP, which enables transparent examination of how first-day ICU variables contribute to predicted outcomes rather than relying solely on black-box predictions [13,14,15]. Across both mortality and length-of-stay models, SHAP analyses revealed clinically coherent patterns that reinforce the face validity of the proposed approaches.

For ICU mortality, the most influential predictors reflected early physiologic instability and organ dysfunction. Reduced 24-h urine output emerged as the strongest contributor to higher predicted mortality risk, highlighting impaired renal perfusion and circulatory compromise early in the ICU course. Markers of respiratory and metabolic derangement, including lower minimum oxygen saturation, higher blood urea nitrogen, and higher anion gap, also ranked prominently, consistent with impaired oxygenation, renal dysfunction, and metabolic stress. Older age at admission and higher maximum temperature further contributed to increased predicted risk, reflecting vulnerability to systemic inflammation and infection. Treatment- and admission-related variables, particularly early exposure to norepinephrine and admission from another hospital, were also associated with higher mortality predictions, capturing hemodynamic instability and greater baseline illness severity. Collectively, these findings align with established determinants of adverse outcomes in critically ill patients and prior atrial fibrillation–focused machine learning studies that emphasize renal, metabolic, and perfusion-related markers of risk [1,4,11].

For ICU length of stay, SHAP analysis of the LightGBM model demonstrated a related but distinct pattern. Longer predicted ICU stays were most strongly associated with higher maximum temperature and early norepinephrine exposure, indicating sustained systemic inflammation and the need for ongoing hemodynamic support. Lower minimum oxygen saturation reduced 24-h urine output, and older age at admission also contributed to prolonged predicted stays, reflecting respiratory compromise and impaired organ perfusion. In addition, laboratory abnormalities, including higher sodium, higher blood urea nitrogen, lower albumin, and higher creatinine, ranked among the most influential predictors, underscoring the role of metabolic imbalance and renal dysfunction in extending ICU duration. Admission from another hospital was additionally associated with longer predicted LOS, likely reflecting greater illness complexity and care transitions.

Although the LOS model explained a smaller proportion of overall variance compared with the mortality model, the directionality and ranking of SHAP-identified predictors provide a coherent and clinically intuitive picture. While mortality risk appears more tightly linked to acute physiologic collapse and shock, prolonged ICU stays reflect sustained organ dysfunction, systemic stress, and resource-intensive care trajectories. This distinction is consistent with prior literature linking atrial fibrillation to both increased mortality risk and longer ICU utilization through overlapping but non-identical clinical pathways [3,4,5,6,16].

### 4.3. Clinical Implications

From a clinical standpoint, the present results have several implications. First, early mortality risk in ICU patients with AF appears to be driven less by the arrhythmia itself and more by the underlying severity of critical illness. This reinforces guideline and consensus recommendations to prioritize hemodynamic stabilization, organ support and treatment of precipitating conditions when managing AF in the ICU [2,3,4,16,18,19]. The SHAP-derived importance of urine output, blood urea nitrogen, creatinine, anion gap and vasoactive support highlights domains that are already central to bedside assessment, suggesting that machine learning models can formalize and quantify patterns that clinicians recognize qualitatively [1,2,3,4,11,13,14,20].

Second, despite explicit strategies to address class imbalance, including class weighting and probability-threshold optimization, the mortality prediction task illustrates a persistent challenge in critical care machine learning. Under low event prevalence, models may achieve reasonable discrimination and risk ranking while still struggling to identify all deaths at clinically acceptable false-positive rates. This reflects the inherent trade-offs associated with rare outcomes rather than a failure of model development. Accordingly, the primary value of such tools may lie in early risk stratification and prioritization rather than stand-alone decision-making [11,13,14,21]. Combining model outputs with established clinical scoring systems, guideline-directed management pathways, and structured multidisciplinary review may therefore yield the greatest clinical benefit.

Third, although the LOS model had limited explanatory power, even approximate estimates of prolonged ICU stay could support resource planning, bed management and family counseling. Prior work has shown that machine learning-based predictions can complement physician judgment and help triage patients who may require extended organ support or early discussions about care goals [11,13,14,21]. In this context, our LOS findings should be viewed as an exploratory rather than a definitive forecasting tool.

### 4.4. Web-Based Implementation and Future Directions

An additional contribution of this study is the deployment of the final XGBoost mortality model and LightGBM LOS model in an online, interactive web application. The tool demonstrates that complex pipelines that incorporate grouped cross-validation, probability estimation, and SHAP-based interpretability can be wrapped into a user-facing interface that accepts first-day ICU predictors and returns individualized risk estimates. This aligns with broader efforts to translate machine learning models into point-of-care decision support and to explore clinician-facing interfaces for AF management [11,14,22,23].

A practical limitation of this work relates to model implementation. The current web-based prototype requires manual entry of all 54 first-day ICU variables, which is not feasible for routine clinical use and may introduce user errors. In modern ICU environments, these data elements, such as demographics (registration systems), vital signs (bedside monitors), laboratory results (laboratory information systems), medication exposures (CPOE/MAR), and organ-support therapies (structured clinical documentation), are normally extracted automatically from the electronic health record (EHR). The model was intentionally developed using the full 54-variable feature set because this configuration provided the highest predictive performance and preserved clinically essential information. Reducing the model to a smaller subset of predictors may reduce predictive performance and could omit clinically informative physiology; therefore, future work should evaluate parsimonious feature sets alongside EHR-integrated automation. Once implemented, such integration would both eliminate manual data entry and enable even richer patient-level predictions by incorporating the full breadth of demographic, physiologic, and medical history data available in the EHR.

### 4.5. Limitations

This study has several limitations that should be considered when interpreting the findings.

First, all data were derived from a single data resource (MIMIC-IV for model development and MIMIC-III for temporal external validation) originating from the same healthcare system. Although we performed rigorous grouped cross-validation and external validation across two distinct time periods and database versions, MIMIC-IV (2008–2019) is newer than MIMIC-III (2001–2012). Therefore, the external evaluation represents a backward-in-time temporal validation (training on newer data and validating on older data), and changes in clinical practice over time may influence performance in either direction. In addition, the development cohort was predominantly White (73%), and performance for under-represented groups (e.g., Black 7.4% and Asian 2.7%) was not evaluated separately; thus, predictive accuracy and fairness across ethnic groups remain uncertain. The models may not generalize to institutions with different case-mix, practice patterns, or documentation conventions [5,6,7,11,21]. Prospective validation in independent, multicenter cohorts will be essential before any clinical implementation [21,22,23]. Future work should evaluate simple post hoc recalibration (e.g., isotonic regression) on external cohorts to improve absolute risk estimates.

Second, the analysis is retrospective and based on routinely collected electronic health record data, which are subject to misclassification, missingness and documentation variability. Medication timing and dosing, nuances of organ support and unmeasured confounders, for example frailty, pre-ICU functional status or ceiling-of-care decisions, are not fully captured. Although we used standardized preprocessing pipelines with consistent imputation across development and validation cohorts, residual bias from data quality issues cannot be excluded [11,13,21].

Third, class imbalance substantially affected the mortality prediction task. Although imbalance-aware strategies were incorporated, including class weighting and threshold optimization, non-survivors represented fewer than 10% of ICU episodes, resulting in reduced sensitivity and low F1 scores at conventional decision thresholds. More advanced imbalance-handling approaches (e.g., specialized loss functions or fully optimized cost-sensitive learning) were not implemented as part of the primary modeling strategy; resampling methods were evaluated only as sensitivity analyses and were not pursued for model optimization [13,14,15,21]. While artificially balancing mortality outcomes can increase apparent recall or F1 scores, such approaches may distort prevalence-dependent decision thresholds and reduce clinical realism; therefore, imbalance-aware evaluation and threshold optimization were prioritized over forced class balancing. Similarly, ICU length-of-stay prediction proved challenging. Although LOS models were temporally externally evaluated on the MIMIC-III cohort, the LightGBM regressor achieved only a small positive R^2^, indicating that many key determinants of ICU stay, such as evolving clinical trajectories, bed availability, discharge planning, and post-ICU care coordination, are not captured by structured variables from the first 24 h of admission. This observation is consistent with prior ICU outcome and LOS literature, which emphasizes that ICU length of stay is strongly influenced by downstream clinical and operational factors beyond early physiologic measurements [6,16,21].

Fourth, predictors were restricted to structured variables available within the first 24 h of ICU admission to support early risk stratification. Excluding ICU stays shorter than 24 h may have preferentially selected patients with sufficient data availability for first-day modeling and may limit applicability to hyperacute ICU presentations. This design supports early risk stratification but does not capture dynamic changes later in the ICU stay. Prior work suggests that incorporating high-frequency time-series data, waveform features, or longitudinal trends can improve the prediction of deterioration and mortality, including approaches that provide visual attribution for multivariate clinical time series [17]. Related studies have also applied explainable machine learning to ICU mortality prediction using static clinical features with SHAP-based interpretation on MIMIC data [24]. The present models should therefore be viewed as early static risk tools rather than dynamic, continuously updating systems.

Fifth, atrial fibrillation was identified using hospital discharge ICD codes, reflecting routine clinical documentation practices. This approach does not permit reliable differentiation between pre-existing atrial fibrillation and new-onset atrial fibrillation during the ICU stay; accordingly, the present analysis focuses on prognostic modeling across a broad AF ICU population rather than subtype-specific trajectories. Future studies incorporating rhythm annotations or time-stamped diagnostic data may enable more granular characterization of AF subtypes. Because AF identification relied on discharge coding, the models are intended for prognostic stratification after AF is identified rather than for prospective AF detection.

Sixth, although SHAP enhances transparency, the underlying XGBoost and LightGBM models remain complex ensemble methods. Some clinicians may still perceive them as black boxes, and SHAP explanations themselves can be misinterpreted without training. We did not evaluate how clinicians understand or use SHAP-based outputs, nor did we assess the impact of the models on decision-making or patient outcomes. As emphasized in recent reviews, careful attention to explainability, workflow integration and user-centered design is essential for safe and effective adoption of machine learning in clinical care [11,13,15,22].

Finally, the web-based application is intended as an educational and exploratory prototype rather than a validated clinical decision support tool. It is not integrated with any live EHR, has not undergone prospective usability, safety, or impact testing, and should not be used to guide individual treatment decisions until such evaluation and appropriate regulatory and governance processes are completed [21,22,23].

## 5. Conclusions

This study used large real-world ICU data from MIMIC-IV and MIMIC-III to develop and validate machine learning models using temporal external validation for ICU mortality and length of stay in patients with atrial fibrillation. An XGBoost classifier provided moderate discrimination for ICU mortality using only first-day clinical data, while a LightGBM regressor showed that first-day structured data provide an insufficient signal to accurately predict ICU LOS (R^2^ = 0.038), likely reflecting downstream clinical and operational factors. SHAP analysis highlighted clinically plausible predictors, including early vasopressor use, renal dysfunction, metabolic derangement, oxygenation, and admission pathways, supporting the clinical face validity of the models and helping translate numerical risk estimates into interpretable clinical insights.

The deployment of these models in a web-based application illustrates how complex machine learning pipelines can be wrapped into a user-facing interface for educational and exploratory use. Although this work has inherent limitations related to retrospective design, single health-system data, and class imbalance, the consistent performance of the mortality model under temporal external validation and the emphasis on transparent interpretability strengthen confidence in the findings. Within this framework, the present results support a potential role for machine learning in improving early prognostication in ICU patients with atrial fibrillation, informing resource planning, and providing a foundation for future dynamic, EHR-integrated decision-support tools embedded within routine critical care workflows.

## Figures and Tables

**Table 1:** Baseline characteristics of ICU patients with atrial fibrillation in the MIMIC-IV development cohort, stratified by survival status.

Variables	All (n = 20,058)	Survivors (n = 18,265)	Non-Survivors (n = 1793)	*p* Value
**Gender, n (%)**				0.004
F	8109 (40.4)	7327 (40.1)	782 (43.6)	
M	11,949 (59.6)	10,938 (59.9)	1011 (56.4)	
**Race, n (%)**				<0.001
American Indian/Alaska Native	37 (0.2)	34 (0.2)	3 (0.2)	
Asian	537 (2.7)	491 (2.7)	46 (2.6)	
Black	1484 (7.4)	1343 (7.4)	141 (7.9)	
Hispanic/Latino	458 (2.3)	413 (2.3)	45 (2.5)	
Native Hawaiian/Other PI	27 (0.1)	23 (0.1)	4 (0.2)	
Other	597 (3.0)	556 (3.0)	41 (2.3)	
Unknown	2272 (11.3)	1938 (10.6)	334 (18.6)	
White	14,646 (73.0)	13,467 (73.7)	1179 (65.8)	
**Primary language, n (%)**				0.064
English	18,097 (90.2)	16,502 (90.3)	1595 (89.0)	
Non-English	1961 (9.8)	1763 (9.7)	198 (11.0)	
**Insurance, n (%)**				<0.001
Medicaid	1472 (7.3)	1356 (7.4)	116 (6.5)	
Medicare	14,958 (74.6)	13,560 (74.2)	1398 (78.0)	
Other	262 (1.3)	240 (1.3)	22 (1.2)	
Private	3264 (16.3)	3037 (16.6)	227 (12.7)	
**Admission type, n (%)**				<0.001
Elective	1211 (6.0)	1185 (6.5)	26 (1.5)	
Emergency/ED	9814 (48.9)	8785 (48.1)	1029 (57.4)	
Observation/other	3060 (15.3)	2787 (15.3)	273 (15.2)	
Surgical same-day admission	2035 (10.1)	2007 (11.0)	28 (1.6)	
Urgent	3938 (19.6)	3501 (19.2)	437 (24.4)	
**Admission location, n (%)**				<0.001
Emergency room	6722 (33.5)	6047 (33.1)	675 (37.6)	
Other	1175 (5.9)	1057 (5.8)	118 (6.6)	
Physician referral/clinic	5874 (29.3)	5587 (30.6)	287 (16.0)	
Transfer from another hospital	5843 (29.1)	5179 (28.4)	664 (37.0)	
Transfer from skilled nursing facility	444 (2.2)	395 (2.2)	49 (2.7)	
Age at admission, years, Median (IQR)	75 (67–83)	75 (66–83)	77 (69–85)	<0.001
**Vasoactive/inotrope use, first 24 h, n (%)**				
Norepinephrine use, first 24 h	3721 (18.6)	2954 (16.2)	767 (42.8)	<0.001
Vasopressin use, first 24 h	1043 (5.2)	712 (3.9)	331 (18.5)	<0.001
Any vasoactive/inotrope use, first 24 h	8106 (40.4)	7088 (38.8)	1018 (56.8)	<0.001
**Key labs and vitals, first 24 h, Median (IQR)**				
Minimum MAP, mmHg	58 (51–64)	58 (52–64)	54 (46–61)	<0.001
Minimum SpO_2_, %	92 (90–94)	92 (90–94)	91 (86–94)	<0.001
Maximum heart rate, beats/min	102 (88–121)	101 (88–119)	114 (94–132)	<0.001
Maximum WBC, 10^9^/L	12.7 (9.2–17.5)	12.6 (9.1–17.2)	14.9 (10.5–20.9)	<0.001
Maximum BUN, mg/dL	25 (17–41)	24 (17–39)	38 (25–58.25)	<0.001
Maximum creatinine, mg/dL	1.2 (0.9–1.9)	1.1 (0.9–1.8)	1.8 (1.1–2.9)	<0.001
Minimum bicarbonate, mmol/L	22 (19–25)	22 (20–25)	20 (16–23)	<0.001
Urine output, first 24 h, mL	1410 (870–2160)	1460 (925–2200)	880 (389–1580)	<0.001
**Medications, first 24 h, n (%)**				
Heparin use, first 24 h	11,600 (57.8)	10,382 (56.8)	1218 (67.9)	<0.001
Aspirin use, first 24 h	10,070 (50.2)	9392 (51.4)	678 (37.8)	<0.001
Amiodarone use, first 24 h	2820 (14.1)	2484 (13.6)	336 (18.7)	<0.001
Esmolol use, first 24 h	412 (2.1)	371 (2.0)	41 (2.3)	0.522
Metoprolol tartrate use, first 24 h	8750 (43.6)	8135 (44.5)	615 (34.3)	<0.001
Warfarin use, first 24 h	1812 (9.0)	1701 (9.3)	111 (6.2)	<0.001
Diltiazem use, first 24 h	2142 (10.7)	1962 (10.7)	180 (10.0)	0.379
Labetalol use, first 24 h	930 (4.6)	846 (4.6)	84 (4.7)	0.966
Metoprolol succinate XL use, first 24 h	1204 (6.0)	1144 (6.3)	60 (3.3)	<0.001
Digoxin use, first 24 h	1402 (7.0)	1255 (6.9)	147 (8.2)	0.04
Propranolol use, first 24 h	69 (0.3)	64 (0.4)	5 (0.3)	0.778

Abbreviations: ICU: intensive care unit, MAP: mean arterial pressure, SpO_2_: oxygen saturation, WBC: white blood cell count, BUN: blood urea nitrogen, Min: minimum value in the first 24 h, Max: maximum value in the first 24 h.

**Table 2:** Temporal external validation performance of ICU mortality classifiers (MIMIC-III). For XGBoost, the prespecified F1-optimized threshold was derived from MIMIC-IV out-of-fold predictions and applied unchanged to MIMIC-III; all other models are reported at the default threshold (0.50).

Model	Threshold Strategy	AUC	AP	Precision	Recall	F1	Balanced Accuracy
XGBoost	F1-optimized (0.18)	0.743	0.226	**0.23**	**0.48**	**0.31**	0.65
Gradient Boosting	Default (0.50)	0.739	0.23	0.31	0.26	0.28	0.6
AdaBoost	Default (0.50)	0.733	0.216	0.36	0.13	0.19	0.55
Logistic Regression	Default (0.50)	0.701	0.201	0.1	0.99	0.18	0.52
Random Forest	Default (0.50)	0.699	0.178	0	0	0	0.5
MLP	Default (0.50)	0.629	0.159	0.22	0.17	0.19	0.55
KNN	Default (0.50)	0.668	0.177	0	0	0	0.5
Decision Tree	Default (0.50)	0.555	0.112	0.16	0.24	0.2	0.56

Abbreviations: AUC: area under the receiver operating characteristic curve, AP: average precision, ICU: intensive care unit, MIMIC-III: Medical Information Mart for Intensive Care III, MLP: multilayer perceptron, KNN: k-nearest neighbors. Note: AUC and AP are threshold-independent. Precision, recall, F1, and balanced accuracy are threshold-dependent. Only the primary XGBoost model underwent probability-threshold optimization using out-of-fold predictions from the MIMIC-IV development cohort. All other models are reported at the default threshold (0.50) to allow consistent algorithmic comparison. Additional Note: Zero recall and F1 values for some models reflect the absence of positive predictions at the default 0.50 threshold under low event prevalence, rather than model implementation errors.

**Table 3:** Temporal external validation performance of regression models for ICU LOS (MIMIC-III).

Model	MAE (h)	RMSE (h)	R^2^
LightGBM	88.9	163.9	0.038
Gradient Boosting	87.2	165.0	0.025
Linear Regression	96.5	164.1	0.036
Random Forest	88.3	168.3	−0.015
KNN	90.6	173.1	−0.072
MLP	146.9	205.7	−0.515

Abbreviations: MAE: mean absolute error, RMSE: root mean square error, R^2^: coefficient of determination, ICU: intensive care unit, LOS: length of stay, LightGBM: light gradient boosting machine, MIMIC-III: Medical Information Mart for Intensive Care III, KNN: k-nearest neighbors, MLP: multilayer perceptron.

## Data Availability

The data analyzed in this study are publicly available from PhysioNet: MIMIC-IV (version 3.1) and MIMIC-III (version 1.4). Access requires completion of required training and acceptance of the PhysioNet credentialed data use agreement. The code used for data extraction and analysis is available from the corresponding author upon reasonable request.

## Supplementary Materials

The following supporting information can be downloaded at https://www.mdpi.com/article/10.3390/healthcare14030356/s1. Table S1: Temporal external validation performance of ICU mortality classifiers at the default probability threshold (0.50); Figure S1: Predicted versus observed ICU length of stay (LOS) for the LightGBM model on MIMIC-III; Figure S2: Histogram of ICU LOS residuals for the LightGBM model on MIMIC-III; Figure S3: Residuals versus predicted ICU length of stay (LOS) for the LightGBM model on MIMIC-III.

## Abbreviations

The following abbreviations are used in this manuscript:

AF	Atrial fibrillation
AP	Average precision
AUC	Area under the receiver operating characteristic curve
BIDMC	Beth Israel Deaconess Medical Center
BUN	Blood urea nitrogen
CV	Cross-validation
EHR	Electronic health record
ICU	Intensive care unit
KNN	k-nearest neighbors
LGBM/LightGBM	Light Gradient Boosting Machine
LOS	Length of stay
MAE	Mean absolute error
MAP	Mean arterial pressure
MIMIC	Medical Information Mart for Intensive Care
ML	Machine learning
MLP	Multilayer perceptron
PR	Precision–recall
R^2^	Coefficient of determination
RMSE	Root mean squared error
ROC	Receiver operating characteristic
SHAP	SHapley Additive exPlanations
SpO_2_	Oxygen saturation
WBC	White blood cell count
XGB/XGBoost	Extreme Gradient Boosting
