# Supplementary material for: Machine Learning Prediction of ICU Mortality and Length of Stay in Atrial Fibrillation: A MIMIC-IV/MIMIC-III Study
Source: Healthcare (Basel). 2026 Jan 30;14(3):356. doi: 10.3390/healthcare14030356 (PMC12897186; doi:10.3390/healthcare14030356)
Supplement: Supplementary file 1 [file healthcare-14-00356-s001.zip › healthcare-4105358-supplementary.pdf]

Table S1. Temporal external validation performance of ICU mortality classifiers at the default probability threshold (0.50).

Temporal external cohort: MIMIC-III
Threshold strategy: Fixed threshold = 0.50 for all models

Model	Threshold	AUC	AP	Precision	Recall	F1	Balanced Accuracy	Accuracy
**XGBoost**	0.50	0.743	0.226	0.00	0.00	0.00	0.50	0.90
**Gradient Boosting**	0.50	0.739	0.230	0.31	0.26	0.28	0.60	0.90
**AdaBoost**	0.50	0.733	0.216	0.36	0.13	0.19	0.55	0.90
**Logistic Regression**	0.50	0.701	0.201	0.10	0.99	0.18	0.52	0.12
**Random Forest**	0.50	0.699	0.178	0.00	0.00	0.00	0.50	0.90
**MLP**	0.50	0.629	0.159	0.22	0.17	0.19	0.55	0.89
**KNN**	0.50	0.668	0.177	0.00	0.00	0.00	0.50	0.90
**Decision Tree**	0.50	0.555	0.112	0.16	0.24	0.20	0.56	0.88

Abbreviations: AUC: area under the receiver operating characteristic curve, AP: average precision, ICU: intensive care unit, MIMIC-III: Medical Information Mart for Intensive Care III, XGBoost: extreme gradient boosting, MLP: multilayer perceptron, KNN: k-nearest neighbors.

Note: All metrics were evaluated on the temporal external MIMIC-III cohort using a fixed probability threshold of 0.50. AUC and AP are threshold-independent. Precision, recall, F1 score, balanced accuracy, and accuracy are threshold-dependent and illustrate the limitations of default probability cutoffs under severe class imbalance, particularly for rare outcomes such as ICU mortality.

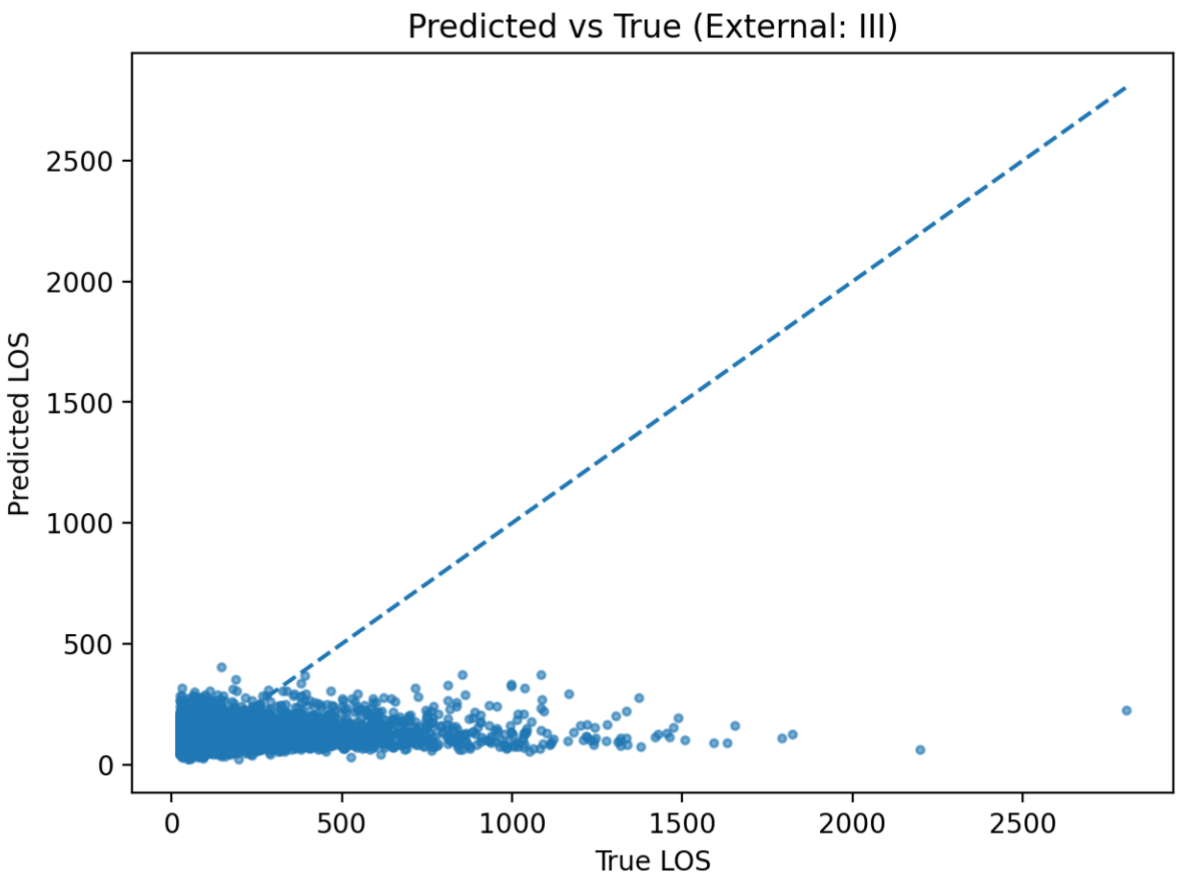

Figure S1. Predicted vs observed ICU LOS (LightGBM, MIMIC-III).

A scatterplot compares predicted ICU length of stay from the LightGBM (Light Gradient Boosting Machine**)** model with observed values in the MIMIC-III cohort, overlaid with the line of identity, highlighting systematic underestimation of very prolonged stays.

Note: LightGBM: light gradient boosting machine, ICU: intensive care unit, LOS: length of stay, MIMIC-III: Medical Information Mart for Intensive Care III.

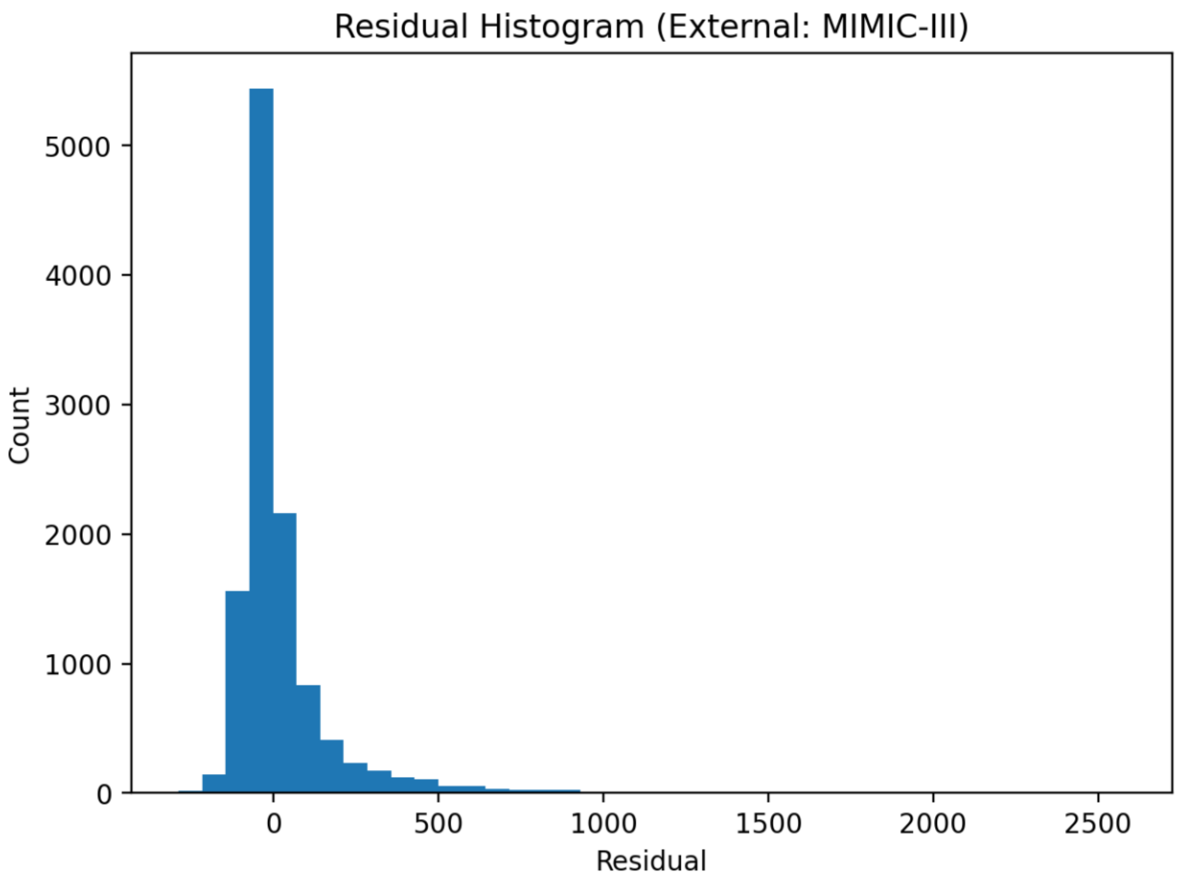

Figure S2. Histogram of LOS residuals (LightGBM, MIMIC-III).

The histogram shows the distribution of residuals (observed minus predicted ICU length of stay) for the LightGBM model in MIMIC-III, demonstrating right-skewed errors with a long tail toward large positive residuals.

Note: LightGBM: light gradient boosting machine, ICU: intensive care unit, LOS: length of stay, MIMIC-III: Medical Information Mart for Intensive Care III.

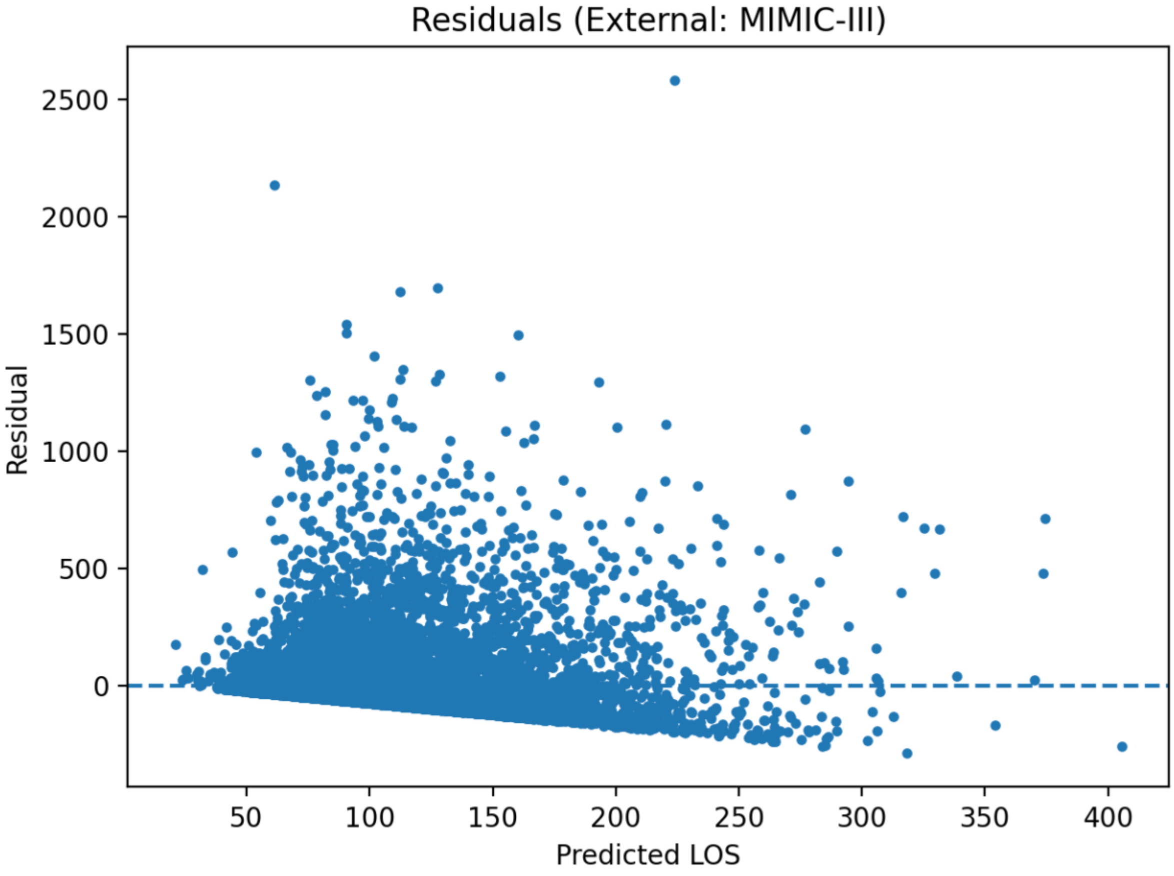

Figure S3. Residuals vs predicted LOS (LightGBM, MIMIC-III).

This scatterplot displays residuals plotted against predicted ICU length of stay for the LightGBM model. Residual variance increases with predicted stay duration, indicating greater uncertainty for patients with longer expected ICU stays.

Note: LightGBM: light gradient boosting machine, ICU: intensive care unit, LOS: length of stay, MIMIC-III: Medical Information Mart for Intensive Care III.
